# Early VGLUT1-specific parallel fiber synaptic deficits and dysregulated cerebellar circuit in the KIKO mouse model of Friedreich ataxia

**DOI:** 10.1242/dmm.030049

**Published:** 2017-12-01

**Authors:** Hong Lin, Jordi Magrane, Elisia M. Clark, Sarah M. Halawani, Nathan Warren, Amy Rattelle, David R. Lynch

**Affiliations:** 1 Departments of Pediatrics and Neurology, The Children's Hospital of Philadelphia, Philadelphia, PA 19104, USA; 2 Feil Family Brain and Mind Research Institute, Weill Cornell Medical College, New York, NY 10065, USA; 3 Perelman School of Medicine, University of Pennsylvania, Philadelphia, PA 19104, USA

**Keywords:** VGLUT1, Parallel fiber synapse, Cerebellar circuit, KIKO, Friedreich ataxia

## Abstract

Friedreich ataxia (FRDA) is an autosomal recessive neurodegenerative disorder with progressive ataxia that affects both the peripheral and central nervous system (CNS). While later CNS neuropathology involves loss of large principal neurons and glutamatergic and GABAergic synaptic terminals in the cerebellar dentate nucleus, early pathological changes in FRDA cerebellum remain largely uncharacterized. Here, we report early cerebellar VGLUT1 (SLC17A7)-specific parallel fiber (PF) synaptic deficits and dysregulated cerebellar circuit in the frataxin knock-in/knockout (KIKO) FRDA mouse model. At asymptomatic ages, VGLUT1 levels in cerebellar homogenates are significantly decreased, whereas VGLUT2 (SLC17A6) levels are significantly increased, in KIKO mice compared with age-matched controls. Additionally, GAD65 (GAD2) levels are significantly increased, while GAD67 (GAD1) levels remain unaltered. This suggests early VGLUT1-specific synaptic input deficits, and dysregulation of VGLUT2 and GAD65 synaptic inputs, in the cerebellum of asymptomatic KIKO mice. Immunohistochemistry and electron microscopy further show specific reductions of VGLUT1-containing PF presynaptic terminals in the cerebellar molecular layer, demonstrating PF synaptic input deficiency in asymptomatic and symptomatic KIKO mice. Moreover, the parvalbumin levels in cerebellar homogenates and Purkinje neurons are significantly reduced, but preserved in other interneurons of the cerebellar molecular layer, suggesting specific parvalbumin dysregulation in Purkinje neurons of these mice. Furthermore, a moderate loss of large principal neurons is observed in the dentate nucleus of asymptomatic KIKO mice, mimicking that of FRDA patients. Our findings thus identify early VGLUT1-specific PF synaptic input deficits and dysregulated cerebellar circuit as potential mediators of cerebellar dysfunction in KIKO mice, reflecting developmental features of FRDA in this mouse model.

## Abstract (precis)

**Summary:** Early VGLUT1-specific parallel fiber synaptic deficits and dysregulated cerebellar circuit might be potential mediators of cerebellar dysfunction and ataxia in the KIKO mouse model of Friedreich ataxia.

## INTRODUCTION

Friedreich ataxia (FRDA) is the most common form of hereditary ataxia, affecting ∼5000 people in the USA. Most affected individuals inherit two alleles containing expanded GAA repeats in intron 1 of the frataxin (*FXN*) gene, resulting in chromatin condensation and reduced expression of the mitochondrial protein frataxin (Bidichandani et al., 1998; Campuzano et al., 1996; Chutake et al., 2014; Grabczyk and Usdin, 2000). This protein is associated with iron-sulfur cluster formation and ATP production (Chiang et al., 2016; Lu and Cortopassi, 2007; Martelli and Puccio, 2014; Parent et al., 2015; Rötig et al., 1997; Vaubel and Isaya, 2013). Symptoms begin as early as 5 years old and worsen over time, with an initial presentation of ataxia, absent lower limb reflexes, and loss of position and vibration sense. This pattern of neurodegeneration results in progressive ataxia, dysmetria and dysarthria (Lynch et al., 2012; Lynch and Seyer, 2014; Pandolfo, 2009). Postmortem studies show degeneration of the large sensory neurons and their axons in the spinal cord, while the cerebellum overtly degenerates to a lesser extent, largely within the dentate nucleus (DN) (Koeppen et al., 2011a,b, 2009).

While the cerebellar cortex is viewed as being spared and the DN is viewed as a key site of FRDA pathology (Koeppen et al., 2011a, 2015), Purkinje cell injury and remodeling has also been reported in postmortem brain samples of FRDA patients (Kemp et al., 2016). However, in most cases, brain samples are available from late-stage individuals who presented with the disease for >30 years. Any evidence of early pathological changes might be lost in the ensuing years of disease progression. FRDA mouse models can be useful tools to search for the earliest pathological changes during disease progression. Complete frataxin knockout is lethal prenatally, and neuron-specific knockouts have an early onset phenotype that resembles the fully developed changes of FRDA (Cossee et al., 2000; Simon et al., 2004); however, this might be too severe to search for the earliest features of the disorder, owing to complete knockout of frataxin, whereas GAA expansion in FRDA decreases frataxin levels to 2-20% of those in controls (Lazaropoulos et al., 2015; Sahdeo et al., 2014). Identification of early changes requires a model in which the phenotype is present, but slowly evolving, in the same manner as FRDA patients progress.

The frataxin knock-in/knockout (KIKO) mouse model of FRDA meets these features. It has a knock-in-expanded GAA repeat on one allele (230 GAAs) and a knockout of *FXN* on the other allele, leading to mice with moderate overall deficiency of frataxin early in life (20-30% of control levels), comparable to the levels in mildly affected patients. No overt neuronal loss appears in initial studies but mRNA panels from tissue share many features with those from patients (Miranda et al., 2002). Recent neurobehavioral studies in KIKO mice show cerebellar ataxia, decreased peripheral sensitivity, and decreased motor strength and endurance at 9 months of age, resembling clinical manifestations observed in late-onset FRDA patients (McMackin et al., 2016). This phenotypically abnormal mouse with solid biochemical deficits, but no overt cell loss, thus provides a model to search for CNS abnormalities that mediate the earliest cerebellar features of FRDA. In the current study, we utilized KIKO mice to search for the early pathological changes in mouse cerebellum at asymptomatic ages.

## RESULTS

### Frataxin is highly expressed in cerebellar Purkinje neurons and DN large principal neurons of C57BL/6 wild-type mice

**Fig. 1.**
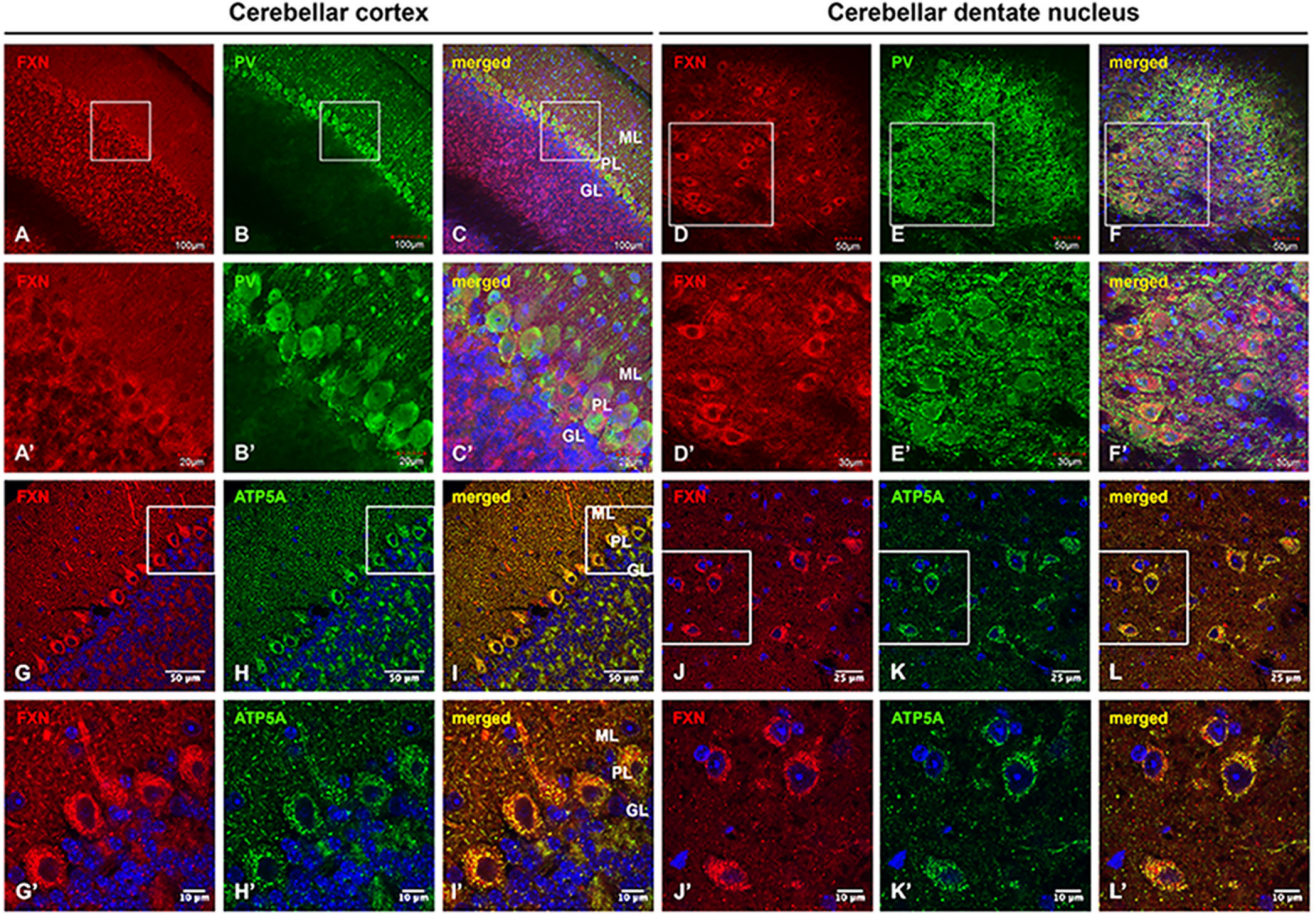
**Frataxin expression and distribution in mouse cerebellum.** (A-F′) Confocal images of frataxin (FXN, red) and PV (green) fluorescence, and merged images with DAPI-stained nuclei (blue), showing wide distribution of FXN in the cerebellar cortex (A-C′) and DN (D-F′) of C57BL/6 normal mice. (G-L′) FXN (red) and mitochondrial marker ATP5A (green) fluorescence, and merged images with DAPI-stained nuclei (blue), showing colocalization of FXN with ATP5A in the cerebellar cortex (G-I′) and DN (J-L′) of C57BL/6 normal mice. Scale bars as indicated.

We first examined the expression and distribution of frataxin in C57BL/6 wild-type mouse cerebellum using double immunohistochemical staining with anti-PV and anti-frataxin antibodies. In mouse cerebellar cortex, PV immunoreactivity is abundant in Purkinje neurons and other interneurons in the molecular layer (ML) (Fig. 1B,B′), while frataxin immunoreactivity (Fig. 1A,A′) is widely distributed in the ML, Purkinje layer (PL) and granular layer (GL), with a high level of expression of frataxin in PV-positive Purkinje neurons (Fig. 1A-C,A′-C′). In the cerebellar DN, frataxin is highly expressed in the large principal neurons surrounded by PV-positive synapses (Fig. 1D-F,D′-F′).

We then examined the expression and distribution of frataxin in relation to mitochondria in mouse cerebellum using double immunohistochemical staining with antibodies against ATP5A (ATP5A1), as a mitochondrial marker, and frataxin. In mouse cerebellar cortex, ATP5A immunoreactivity is widely distributed in the ML, PL and GL, with a high level of expression in the soma and dendrites of Purkinje neurons (Fig. 1H,H′). Frataxin immunoreactivity (Fig. 1G,G′) colocalizes with ATP5A immunoreactivity (Fig. 1H,H′) in mouse cerebellar cortex (Fig. 1I,I′). In cerebellar DN, ATP5A immunoreactivity (Fig. 1K,K′) is highly expressed in large principal neurons and colocalizes with frataxin immunoreactivity (Fig. 1J,J′) in the principal neurons (Fig. 1L,L′). This suggests crucial roles of frataxin in maintaining cerebellar mitochondrial function under normal physiological conditions.

### Frataxin and the mitochondrial marker ATP5A are more abundant in VGLUT1-positive glutamatergic synaptic terminals than in GAD65-positive GABAergic synaptic terminals in wild-type mouse cerebellar cortex

**Fig. 2.**
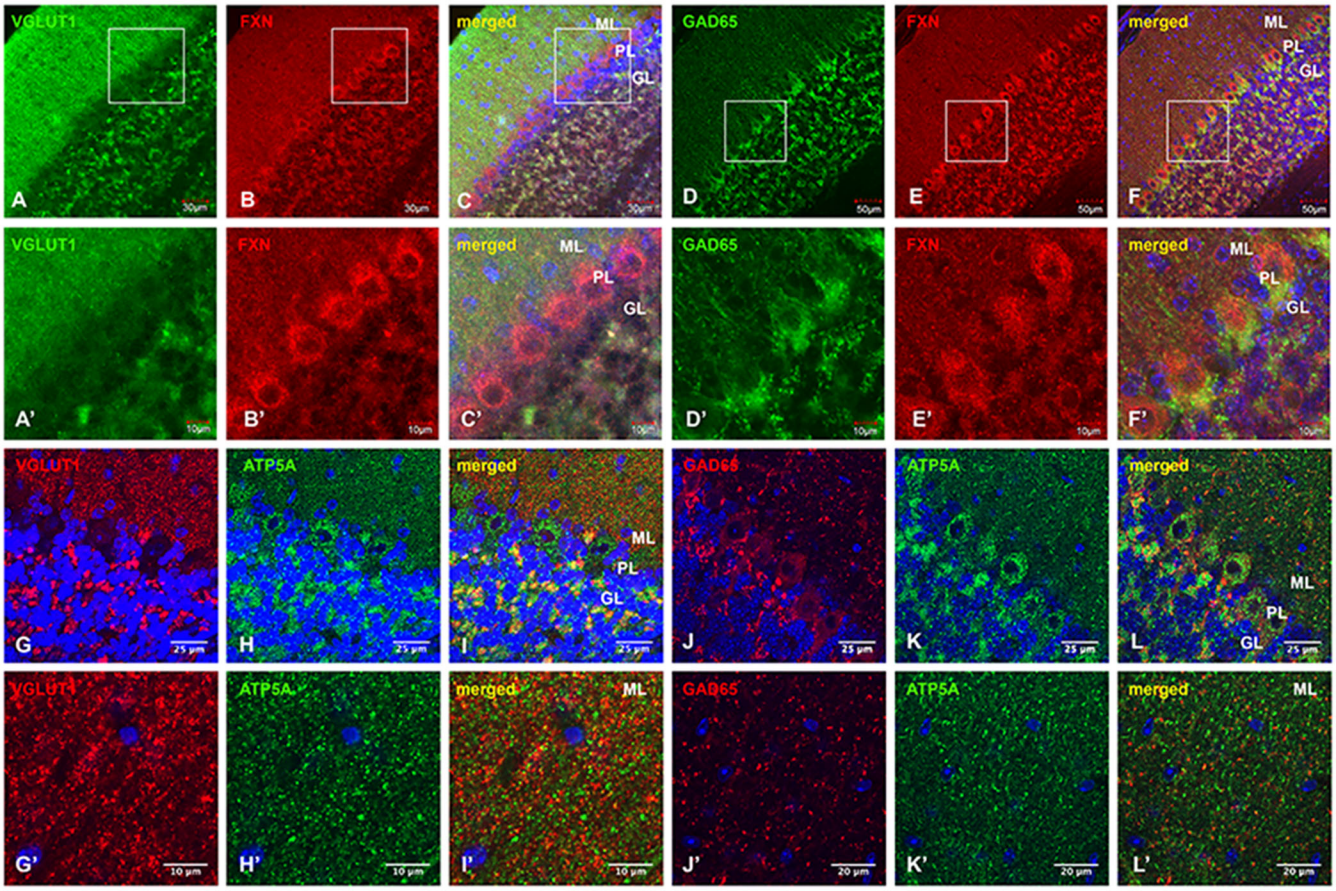
**Frataxin and the mitochondrial marker ATP5A are more abundant in VGLUT1-positive glutamatergic synaptic terminals than in GAD65-positive GABAergic synaptic terminals in C57BL/6 mouse cerebellar cortex.** (A-C′) Confocal images of VGLUT1 (green) and FXN (red) fluorescence, and merged images with DAPI-stained nuclei (blue), showing FXN in VGLUT1-positive glutamatergic synaptic terminals. (D-F′) GAD65 (green) and FXN (red) fluorescence, and merged images with DAPI-stained nuclei (blue), showing GAD65-positive GABAergic synaptic terminals. (G-I′) VGLUT1 (red) and ATP5A (green) fluorescence, and merged images with DAPI-stained nuclei (blue), showing ATP5A in VGLUT1-positive glutamatergic synaptic terminals. (J-L′) GAD65 (red) and ATP5A (green) immunofluorescence, and merged images with DAPI-stained nuclei (blue), in GAD65-positive GABAergic synaptic terminals. Scale bars as indicated.

We further examined the distribution of frataxin in glutamatergic and GABAergic synaptic terminals of the cerebellar cortex in wild-type C57BL/6 mice using double immunohistochemical staining with anti-frataxin and anti-VGLUT1 (SLC17A7) or anti-GAD65 (GAD2) antibodies. VGLUT1 immunoreactivity is most prominent in the ML, with lower intensity in the GL (Fig. 2A,A′). Frataxin immunoreactivity (Fig. 2B,B′) colocalizes with VGLUT1-positive glutamatergic synaptic terminals in the ML and GL (Fig. 2C,C′). By contrast, frataxin immunoreactivity (Fig. 2E,E′) does not colocalize with GAD65-positive GABAergic synaptic terminals (Fig. 2D,D′) in the ML and GL (Fig. 2F,F′).

We then examined the expression and distribution of mitochondria in relation to frataxin, glutamatergic and GABAergic synaptic terminals in mouse cerebellar cortex using double immunohistochemical staining with anti-ATP5A and anti-VGLUT1 or anti-GAD65 antibodies. While there is more colocalization of ATP5A immunoreactivity (Fig. 2H,H′) with VGLUT1-positive glutamatergic synaptic terminals (Fig. 2G,G′) in the ML and GL (Fig. 2I,I′), there is much less colocalization of ATP5A immunoreactivity (Fig. 2K,K′) with GAD65-positive GABAergic synaptic terminals (Fig. 2J,J′) in the ML and GL (Fig. 2L,L′). Taken together, our findings indicate that frataxin and the mitochondrial marker ATP5A are more abundant in VGLUT1-positive glutamatergic synaptic terminals than in GAD65-positive GABAergic synaptic terminals of wild-type mouse cerebellar cortex, suggesting that frataxin and mitochondria have more important roles in VGLUT1-containing glutamatergic synaptic terminals than in GAD65-containing GABAergic terminals.

### Early specific reduction of VGLUT1 levels in cerebellum of frataxin KIKO mice at asymptomatic ages

**Fig. 3.**
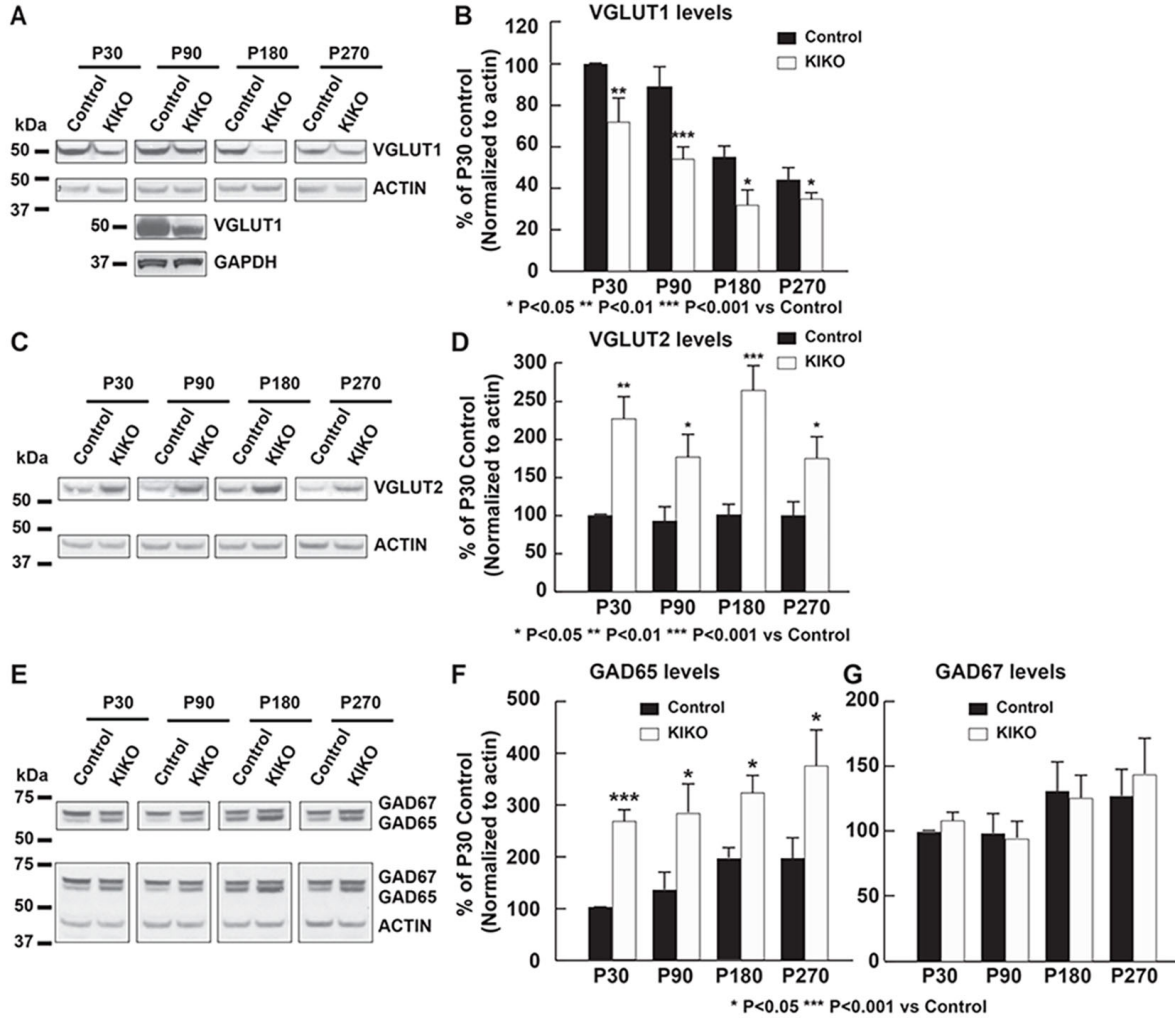
**Specific reduction of VGLUT1 levels and increased VGLUT2 and GAD65 levels in cerebellar homogenates of frataxin-deficient KIKO mice at asymptomatic and potentially symptomatic ages.** Western blot and quantification analysis of cerebellar homogenates (30 μg per lane) showing (A,B) VGLUT1, (C,D) VGLUT2 or (E-G) GAD65/67 levels, with actin or GAPDH as internal control, in KIKO mice and age-matched controls at postnatal days P30, P90, P180, and P270 (*n*=3-8 for KIKO and control mice per time point). Data are mean±s.e.m. *P<0.05, **P<0.01, ***P<0.001, two-tailed, unpaired Student’s *t*-test. Blots in Fig. 3 were stripped and reprobed with multiple antibodies in Fig. 7 and in figures in Lin et al. (2017); anti-actin and anti-GAPDH served as the loading control for each.

We examined whether frataxin levels are decreased in cerebellar homogenates of KIKO mice at asymptomatic (1, 3 and 6 months old) and potentially symptomatic (9 months) ages (McMackin et al., 2016). As expected, frataxin levels are significantly reduced in cerebellar homogenates of KIKO mice compared with age-matched controls at all ages (16-29% residual frataxin) (Lin et al., 2017). We then searched for early cerebellar synaptic abnormalities in KIKO mice by examining levels of glutamatergic presynaptic markers [VGLUT1 and VGLUT2 (SLC17A6)] and GABAergic synaptic markers [GAD65 and GAD67 (GAD1)] in cerebellar homogenates of KIKO mice at asymptomatic (1, 3 and 6 months old) and potentially symptomatic (9 months) ages (McMackin et al., 2016). In wild-type control mice, VGLUT1 levels in cerebellar homogenates are decreased at P180 and P270 compared with P30 and P90 (Fig. S1A), whereas VGLUT2 (Fig. S1B) and GAD67 (Fig. S1C) remain unaltered, and GAD65 levels are modestly increased at P90, P180 and P270 compared with P30 (Fig. S1C). In KIKO mice, VGLUT1 levels in cerebellar homogenates are significantly decreased at asymptomatic (1, 3 and 6 months old) (28%, 43%, 42% reduction; *P*<0.01, *P*<0.001, *P*<0.05 versus controls, respectively) and potentially symptomatic (9 months old, 21% reduction, *P*<0.05 versus controls) ages (Fig. 3A,B). By contrast, the levels of VGLUT2 are significantly increased at all ages (*P*<0.05, *P*<0.01 and *P*<0.001 versus controls) (Fig. 3C,D). Moreover, similar to VGLUT2, the levels of the GABAergic presynaptic marker GAD65 are significantly increased (*P*<0.05, *P*<0.001 versus controls) (Fig. 3E,F), whereas GAD67 levels remain unaltered (Fig. 3E,G), in cerebellar homogenates of KIKO mice compared with age-matched controls. These findings demonstrate early VGLUT1-specific synaptic input deficits and dysregulated VGLUT2 and GAD65 synaptic inputs in the cerebellum of asymptomatic KIKO mice.

### Early VGLUT1-specific parallel fiber synaptic input deficits in cerebellar ML of asymptomatic KIKO mice

**Fig. 4.**
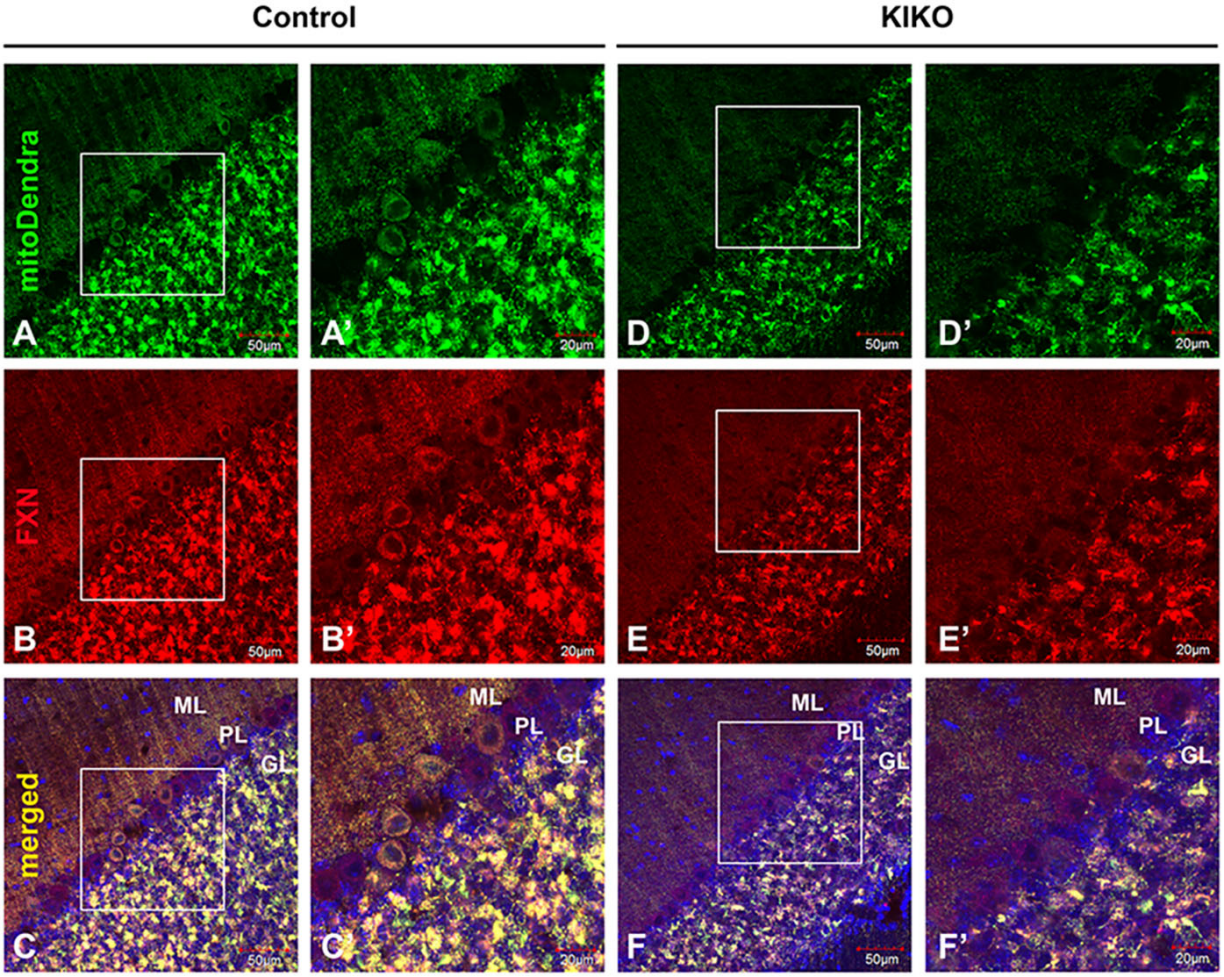
**Reduced frataxin levels in the cerebellum of asymptomatic KIKO mice carrying a mitoDendra marker.** (A-F′) Confocal images of mitoDendra (green) and FXN (red) immunofluorescence, and merged images with DAPI-stained nuclei (blue), showing a reduction in the overall levels of FXN and mitoDendra in the cerebellar cortex of mitoDendra-KIKO mice (D-F,D′-F′) compared with control mice (A-C,A′-C′) at 3 months of age (P90). Scale bars as indicated.

To further pursue the VGLUT1-specific synaptic deficits in KIKO mouse cerebellum, we utilized the KIKO mouse and a transgenic mouse expressing fluorescent Dendra-labeled mitochondria in the nervous system (mitoDendra-KIKO mouse) (Magrané et al., 2014). The mitoDendra transgene allows us to study the exact relationship of mitochondria to the synaptic changes of KIKO mice, and provides a marker of the location of mitochondria relative to detailed cerebellar anatomy. Dendra-labeled mitochondria are widely expressed in the cerebellar cortex of mitoDendra mice (Fig. 4A,A′,D,D′). We observed that frataxin immunoreactivity (Fig. 4B,B′,E,E′) colocalizes with Dendra-labeled mitochondria as expected (Fig. 4C,C′,F,F′). The overall levels of frataxin and mitoDendra are markedly reduced in the cerebellar cortex of mitoDendra-KIKO mice (Fig. 4E,E′) compared with controls (Fig. 4B,B′) at 3 months of age, consistent with frataxin and mitochondrial deficiency in mitoDendra-KIKO mice.

**Fig. 5.**
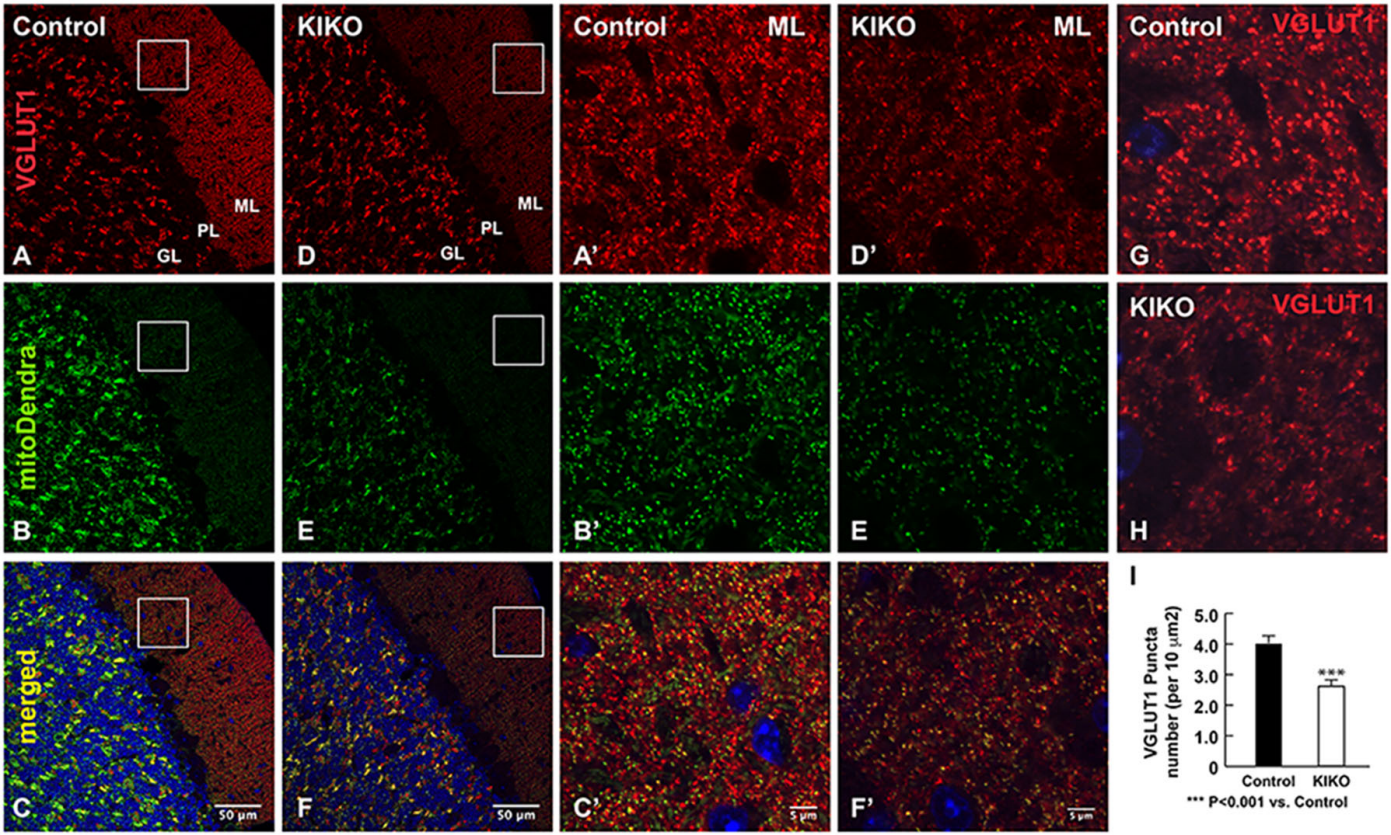
**VGLUT1-specific PF synaptic inputs are impaired in the cerebellar ML of asymptomatic KIKO mice carrying a mitoDendra marker.** (A-F) Confocal images of VGLUT1 (red) and mitoDendra (green) fluorescence, and merged images with DAPI-stained nuclei (blue), showing specific reduction of VGLUT1 immunoreactivity in the cerebellar ML, but not the GL, of P90 mitoDendra-KIKO mice (D-F) compared with age-matched control mice (A-C). (A′-F′) Higher magnification confocal images showing marked reduction of VGLUT1-positive presynaptic terminals in the cerebellar ML of P90 mitoDendra-KIKO mice (D′-F′) compared with control mice (A′-C′). (G,H) Confocal images of VGLUT1-positive presynaptic terminals (red immunofluorescence) in the cerebellar ML of P90 mitoDendra-KIKO mice (H) compared with control mice (G). (I) Quantification of VGLUT1-positive puncta showing significant reduction of VGLUT1-positive presynaptic terminals (*n*=6 sections for two KIKO mice and *n*=9 sections for three controls). Data are mean±s.e.m. ***P<0.001, two-tailed, unpaired Student’s *t*-test. Scale bars as indicated.

We then compared VGLUT1 immunoreactivity in the cerebellum of mitoDendra-KIKO mice with that of age-matched controls at 3 months of age. In the cerebellar cortex of controls, similar to wild-type C57BL/6 mice (Fig. 2), VGLUT1 immunoreactivity is most prominent in the ML (Fig. 5A), in which parallel fibers (PFs) arise from granular cells and make numerous VGLUT1-positive synapses on dendrites of Purkinje neurons. VGLUT1 immunoreactivity is sparser in the GL (Fig. 5A). Interestingly, the overall levels of VGLUT1 immunoreactivity are markedly decreased in the ML, but not the GL, of KIKO mice (Fig. 5D,E) compared with age-matched controls (Fig. 5A-C), suggesting specific deficiency of VGLUT1-containing PF synaptic inputs onto Purkinje neurons. VGLUT1-positive glutamatergic presynaptic terminals are dramatically decreased in the ML of KIKO cerebellum (Fig. 5D′-F′) compared with controls (Fig. 5A′-C′). Quantification of VGLUT1-positive puncta confirms the reduction of VGLUT1-containing presynaptic terminals in the ML of KIKO cerebellum compared with controls (Fig. 5G-I, *P*<0.001). Our findings thus demonstrate early VGLUT1-specific PF synaptic input deficits onto Purkinje neurons in FRDA mouse cerebellum.

**Fig. 6.**
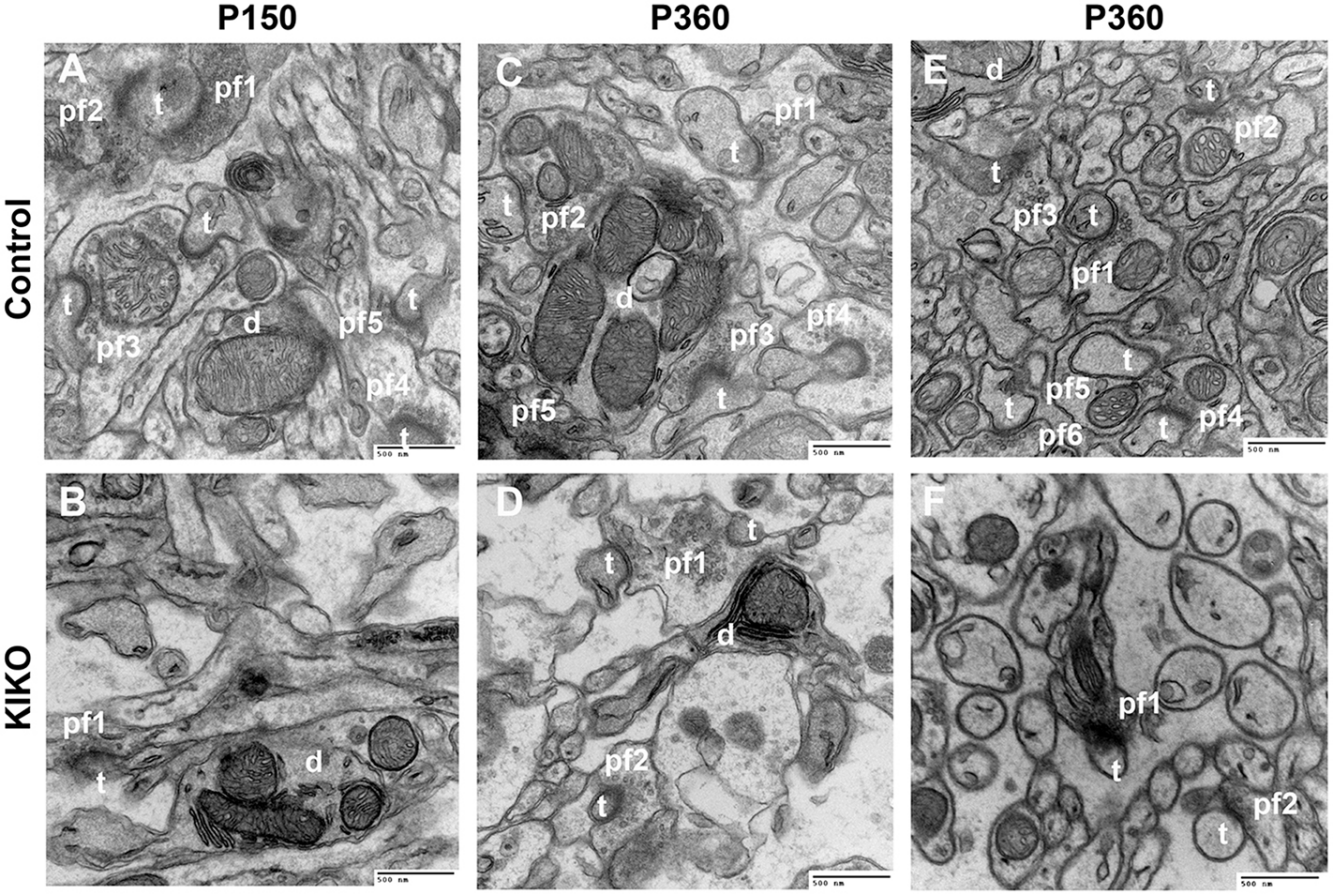
**Impaired ultrastructures of cerebellar ML and PF synaptic deficits on Purkinje neurons in asymptomatic and potentially symptomatic KIKO mice.** (A-F) Electron microscopy images of cerebellar ML in asymptomatic (P150) and potentially symptomatic (P360) KIKO mice (B,D,F) compared with controls (A,C,E). KIKO mice show impaired ultrastructures of cerebellar ML with enlarged empty space, fewer and smaller mitochondria in the dendritic branches (d) of Purkinje neurons, and decreased PF presynaptic terminals (pf) on the thorns (t) of Purkinje neuron dendritic branches in P150 (B) and P360 (D) KIKO mice compared with age-matched control mice (A,C). A high abundance of PF synaptic terminals contain round-shaped mitochondria and form synapses on the thorns of Purkinje neuron dendritic branches in the cerebellar ML of control mice (E) and are markedly reduced in P360 KIKO mice (F). Scale bars as indicated.

To further pursue the association of VGLUT1-specific PF synaptic inputs with cerebellar dysfunction in KIKO mice, we utilized transmission electron microscopy to examine the ultrastructure of PF synaptic terminals on Purkinje neurons in the cerebellar ML at asymptomatic (P150) and potentially symptomatic (P360) ages. In wild-type control mice, the numerous mitochondria are fully packed in the small dendritic branches and spiny branchlets of Purkinje neurons (Fig. 6A,C; Fig. S2A,C) (d, dendritic branches and branchlets), suggesting important physiological roles of mitochondria in Purkinje neuron dendritic function. The abundant PF presynaptic terminals contain round-shaped mitochondria and form synapses on the thorns of Purkinje neuron dendritic branches (Fig. 6A,C,E; Fig. S2A,C) (pf, parallel fiber; t, thorns). In P360 KIKO mice, we observed an overall impaired ultrastructure with an enlarged empty space in the cerebellar ML (Fig. 6D; Fig. S2D) compared with age-matched controls (Fig. 6C; Fig. S2C). The small dendritic branches and spiny branchlets of Purkinje neurons have fewer and smaller mitochondria with disrupted cristae (Fig. 6D; Fig. S2D) compared with those of controls (Fig. 6C; Fig. S2C). Furthermore, the number of PF synapses (pf) in the cerebellar molecular layer is markedly reduced in KIKO mice (Fig. 6D,F; Fig. S2D) compared with controls (Fig. 6C,E; Fig. S2C), demonstrating severe PF synaptic deficits in older KIKO mice. Interestingly, the overall impairment of ultrastructure in the cerebellar ML is also observed in P150 KIKO mice (Fig. 6B; Fig. S2B) compared with age-matched controls (Fig. 6A; Fig. S2A), but to a lesser extent compared with P360 KIKO mice (Fig. 6D; Fig. S2D). Similar reduction of PF synapses occurs in the cerebellar ML compared with controls (Fig. 6A,B; Fig. S2A,B). The impairment of mitochondria is also observed in the small dendritic branches and spiny branchlets of Purkinje neurons in P150 KIKO mice compared with controls (Fig. S2A,B). This suggests that PF synaptic deficits might contribute to cerebellar dysfunction and possibly progressive ataxia in this FRDA model.

### Parvalbumin levels are specifically reduced in cerebellar Purkinje neurons of asymptomatic KIKO mice

**Fig. 7.**
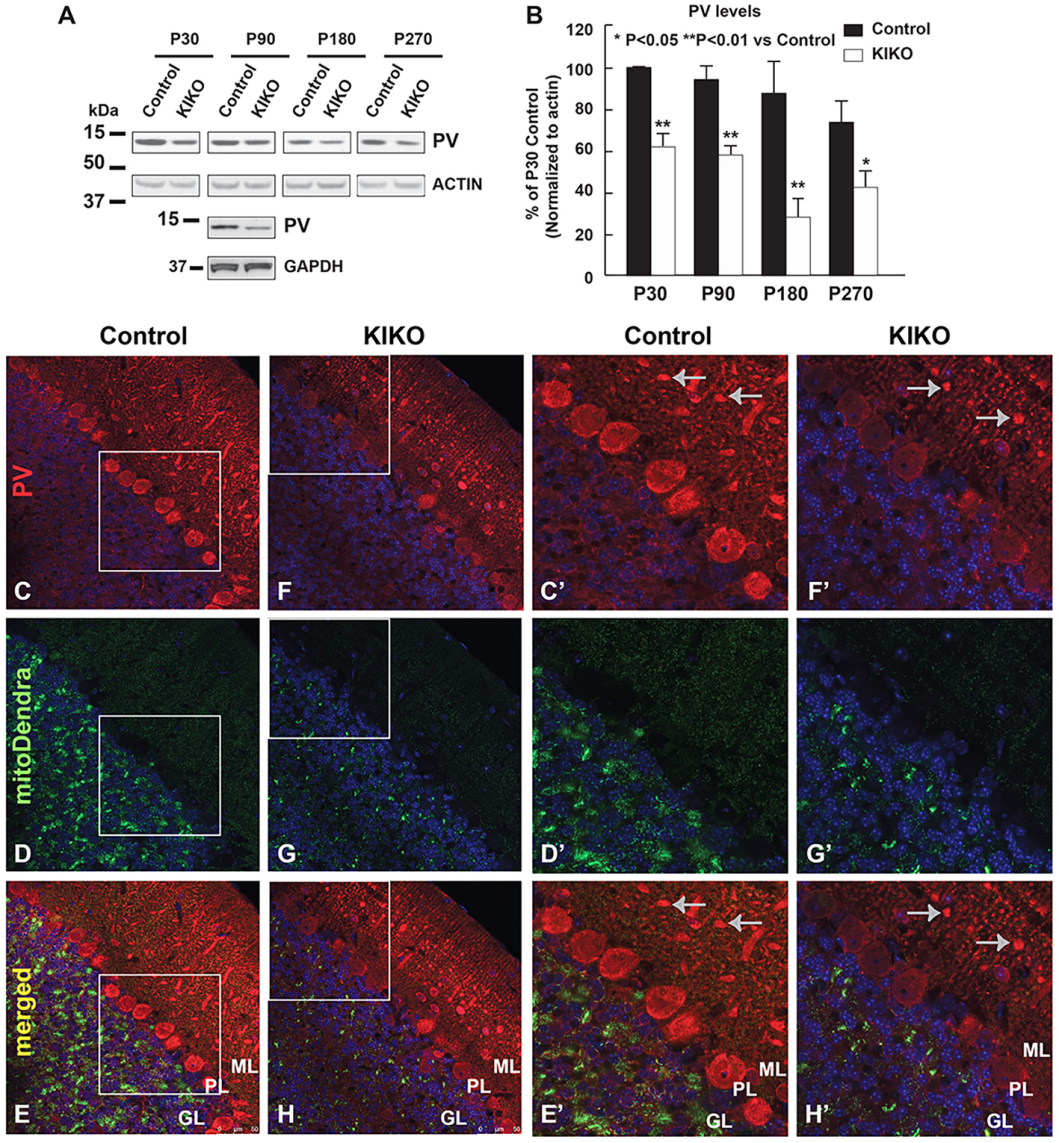
**PV levels are specifically reduced in Purkinje neurons, but preserved in other interneurons of the ML, in asymptomatic KIKO mice.** (A,B) Western blot and quantification analysis of cerebellar homogenates (30 μg per lane) showing PV levels, as well as actin or GAPDH as an internal control, in the cerebellum of KIKO mice and age-matched control mice at P30, P90, P180 and P270 (*n*=3-8 for KIKO and control mice per time point). Data are mean±s.e.m. *P<0.05, **P<0.01, two-tailed, unpaired Student’s *t*-test. Blots in Fig. 7 were stripped and reprobed with multiple antibodies in Fig. 3 and in figures in Lin et al. (2017); anti-actin and anti-GAPDH served as the loading control. (C-H) Confocal images of PV (red) and mitoDendra (green) fluorescence, and merged images with DAPI-stained nuclei (blue), showing marked reduction of PV and mitoDendra in the cerebellar cortex of P90 mitoDendra-KIKO mice (F-H) compared with age-matched control mice (C-E). (C′-H′) Higher magnification confocal images showing marked reduction of PV in Purkinje neurons, but not in other interneurons of the ML (arrows), of P90 mitoDendra-KIKO mice (F′-H′) compared with control mice (C′-E′). Scale bars as indicated.

Parvalbumin (PV) levels frequently reflect neuronal excitatory activity, and PV is highly expressed in cerebellar Purkinje neurons (Patz et al., 2004; Vig et al., 1996). We examined PV levels in cerebellar homogenates of KIKO mice compared with control mice at P30, P90, P180 and P270. In wild-type control mice, PV levels in cerebellar homogenates remain unaltered or slightly decreased at P180 and P270 compared with P30 and P90 (Fig. S1D). In KIKO mice, PV levels are significantly reduced in cerebellar homogenates from P30 to P270 (39-69% reduction, *P*<0.05 and *P*<0.01) (Fig. 7A,B), suggesting PV dysregulation in KIKO cerebellum. In order to identify where changes in PV levels occur, we examined PV immunoreactivity by immunohistochemistry in the cerebellum of mitoDendra-KIKO mice compared with age-matched controls at P90. In the cerebellar cortex of control mice, similar to wild-type C57BL/6 mice (Fig. 1), PV is highly expressed in Purkinje neurons and other interneurons in the ML (Fig. 7C,C′). Remarkably, PV levels are specifically reduced in Purkinje neurons, but preserved in other interneurons of the ML (Fig. 7C′,E′,F′,H′, arrows), in asymptomatic KIKO mice (Fig. 7F-H,F′-H′) compared with age-matched control mice (Fig. 7C-E,C′-E′), demonstrating specific PV deficits in the Purkinje neurons of asymptomatic KIKO mice. The number of Purkinje neurons appears to be unaltered, consistent with Purkinje cell dysfunction rather than neuronal loss in this FRDA mouse model (Kemp et al., 2016). This suggests that PV deficits in Purkinje neurons are downstream from synaptic abnormalities in KIKO cerebellum as PV levels frequently reflect excitatory neuronal activity, implicating early Purkinje cell dysfunction, instead of neuronal loss, in the evolving phenotype of this FRDA mouse model.

### Moderate loss of principal neurons in cerebellar DN of KIKO mice at asymptomatic age

**Fig. 8.**
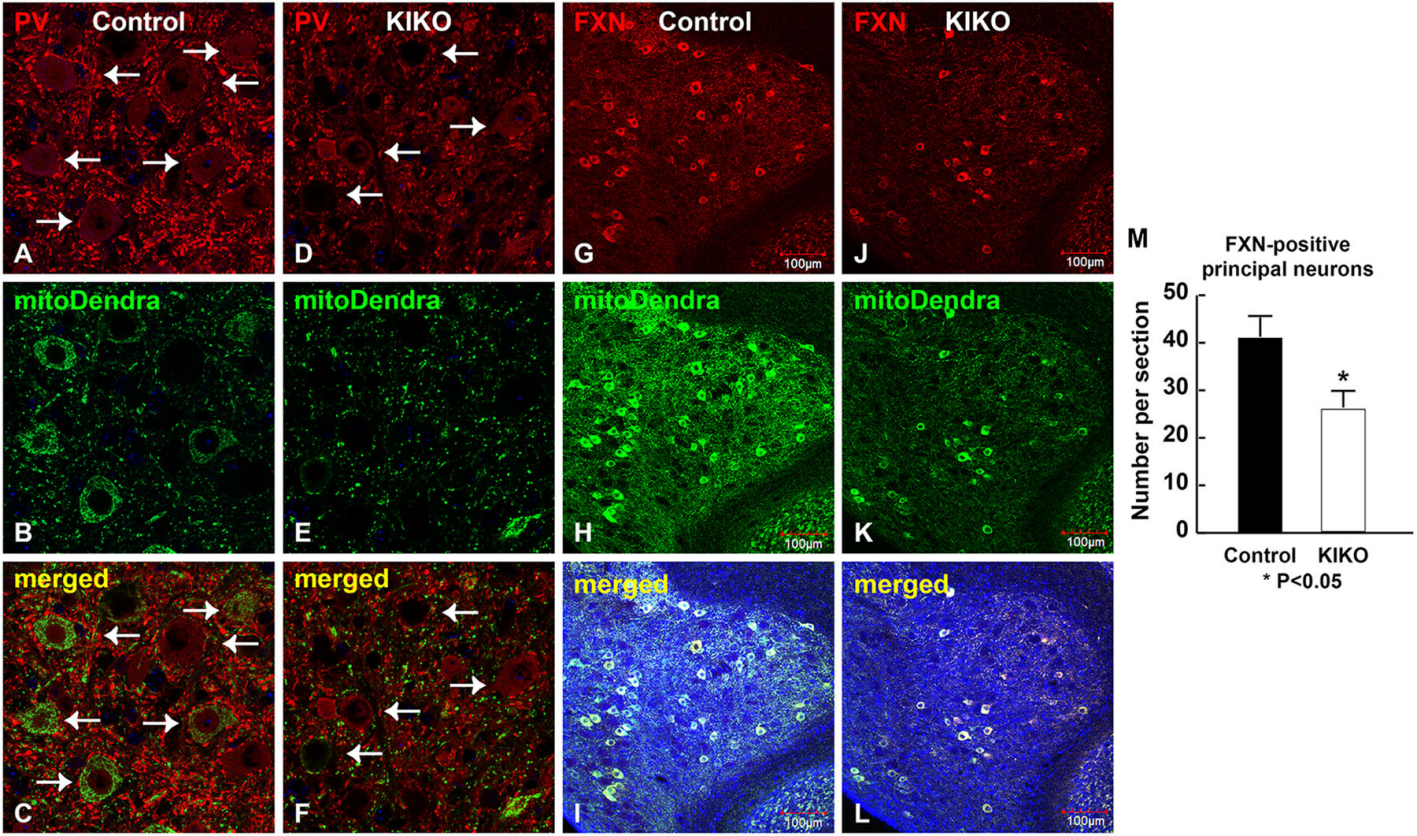
**Moderate loss of large principal neurons in the DN of asymptomatic KIKO mice.** (A-F) Confocal images of PV (red) and mitoDendra (green) fluorescence, and merged images with DAPI-stained nuclei (blue), showing reduction of PV-positive synapses and the surrounding principal neurons in the DN of P90 mitoDendra-KIKO mice (D-F) compared with age-matched controls (A-C). (G-L) Confocal images of FXN (red) and mitoDendra (green) fluorescence, and merged images with DAPI-stained nuclei (blue), showing reduction of large principal neurons in the DN of P90 mitoDendra-KIKO mice (J-L) compared with controls (G-I). (M) Quantification analysis showing a moderate, but significant, decrease in the number of FXN-positive large principal neurons in the DN of mitoDendra-KIKO mice (J) compared with controls (G) (*n*=9 sections for three KIKO mice and three controls). Data are mean±s.e.m. *P<0.05, two-tailed, unpaired Student's *t*-test. Scale bars as indicated.

Cerebellar Purkinje neurons project to the large principal neurons in the DN. We observed reduction of PV-positive synaptic terminals surrounding the large principal neurons in the DN of mitoDendra-KIKO mice (Fig. 8D,F, arrows) compared with control mice (Fig. 8A,C, arrows), consistent with PV reduction in the Purkinje neurons of KIKO mice (Fig. 7). Interestingly, the number of large principal neurons appears to be reduced in the DN of mitoDendra-KIKO mice at P90 (Fig. 8D-F) compared with controls (Fig. 8A-C). We further examined frataxin immunoreactivity in the DN of cerebellum sections. The overall levels of frataxin are decreased in the DN of KIKO mice. In addition, the number of frataxin-positive large principal neurons is moderately but significantly reduced in the DN of mitoDendra-KIKO mice (Fig. 8J-L) compared with control mice at P90 (Fig. 8G-I,M, *P*<0.05), matching the atrophy of large principal neurons in the DN observed in FRDA patients (Koeppen et al., 2011a, 2015). This has not been identified before in this FRDA model, especially at asymptomatic ages, showing the relevance of this model to study neuropathological changes in FRDA.

## DISCUSSION

The present study uses a frataxin-deficient FRDA mouse model (KIKO mouse) to show early VGLUT1-specific PF synaptic input deficits and dysregulated cerebellar circuit at asymptomatic ages. The levels of VGLUT1 and PV are significantly decreased, whereas VGLUT2 and GAD65 levels are significantly increased, in cerebellar homogenates of asymptomatic KIKO mice, suggesting early VGLUT1-specific synaptic deficits and dysregulated VGLUT2 and GAD65 synaptic inputs in KIKO cerebellum. Significant reduction of VGLUT1-positive presynaptic terminals and PF synaptic deficits in the cerebellar ML further demonstrate early VGLUT1-specific PF synaptic deficits in asymptomatic and symptomatic KIKO mice. PV levels are specifically reduced in Purkinje neurons, but preserved in other interneurons of the ML, suggesting specific PV dysregulation in Purkinje neurons. Consistent with DN atrophy in FRDA patients, a moderate loss of large principal neurons is also observed in the DN of asymptomatic KIKO mice (Koeppen et al., 2011a). Our findings thus identify early VGLUT1-specific PF synaptic deficits and dysregulated cerebellar circuit as potential mediators of cerebellar dysfunction, which reflects developmental features of FRDA in this mouse model.

Early synaptic dysfunction and degeneration have been implicated in the pathogenesis of other neurodegenerative diseases including Alzheimer's, Huntington's and Parkinson's diseases (Cai and Tammineni, 2016; Calkins et al., 2011; Onyango et al., 2017; Shirendeb et al., 2012). Glutamatergic and GABAergic synaptic degeneration are found in the cerebellar DN of FRDA postmortem brain samples (Koeppen et al., 2011a, 2015). Our findings demonstrate significant reduction of VGLUT1 levels in KIKO cerebellum at asymptomatic and symptomatic ages. Conversely, VGLUT2 levels are increased in KIKO cerebellum. VGLUT1 is a selective marker for PFs that arise from granule cells and form numerous synapses onto dendrites of Purkinje neurons in the ML (Fremeau et al., 2001). VGLUT2 is a selective marker for climbing fiber (CF) synaptic terminals that project onto Purkinje neurons and undergo supernumerary elimination from multiple- to mono-innervation during development (Fremeau et al., 2001; Hashimoto and Kano, 2013). Proper formation of PF synapses is necessary for eliminating CF innervation on Purkinje cells during development (Hashimoto et al., 2009; Vanni et al., 2015; Watanabe and Kano, 2011). Our findings thus imply that reduction of VGLUT1-containing PFs might lead to abnormal CF synapse elimination and increases in VGLUT2 levels in KIKO cerebellum at early ages. Furthermore, our findings show specific reduction of VGLUT1-positive presynaptic terminals and PF synapses in the cerebellar ML of KIKO mice at asymptomatic and potentially symptomatic ages, demonstrating PF synaptic deficits in KIKO cerebellar cortex at asymptomatic ages. This suggests that early VGLUT1-specific PF synaptic deficits might contribute to abnormal cerebellar circuit development in this FRDA model. Interestingly, our findings not only show imbalanced VGLUT1- and VGLUT2-containing glutamatergic synaptic inputs, but also altered GABAergic synaptic inputs with increased GAD65 levels in KIKO cerebellar homogenates, whereas GAD67 levels remain unaltered. GAD65 is primarily located at GABAergic synaptic terminals and serves as a marker for other interneurons, while GAD67 is primarily located in the soma of GABAergic interneurons and most abundant in Purkinje neurons. Heterologous competition among GABAergic and glutamatergic inputs is important for normal cerebellar circuit development, and GABAergic transmission onto Purkinje cells is a crucial factor for CF synapse elimination (Hashimoto et al., 2009; Nakayama et al., 2012). Our findings thus suggest that increased GAD65 levels could result from early VGLUT1-specfic PF synaptic deficits, which also influence CF synapse elimination during development, leading to an increase of VGLUT2 levels. Our findings thus strongly suggest that abnormal cerebellar synaptic and circuit development in KIKO cerebellum might be potential mediators of cerebellar dysfunction and ataxia in this FRDA model.

Purkinje cells are the most distinctive neurons in the brain as they receive more synaptic inputs than any other type of neuron in the brain. Estimates of the number of spines on a single human Purkinje neuron run as high as 200,000; therefore, synaptic inputs on Purkinje neurons are crucial for cerebellar neuronal functions. Imbalanced glutamatergic and GABAergic synaptic inputs on Purkinje neurons might lead to Purkinje neuronal dysfunction in this FRDA model. Our findings identify early and specific PV reduction in Purkinje neurons at asymptomatic ages. PV is highly expressed in Purkinje neurons and its levels depend on neuronal activity (Patz et al., 2004). PV reduction thus reflects reduced neuronal activity in Purkinje neurons resulting from imbalanced excitatory and inhibitory synaptic inputs in KIKO cerebellum. Moreover, our findings identify moderate loss of large principal neurons in the DN of KIKO cerebellum at asymptomatic ages, consistent with that of FRDA patients (Koeppen et al., 2011a). Taken together, our findings thus suggest that a dysregulated cerebellar circuit including imbalanced glutamatergic and GABAergic synaptic inputs, PV dysregulation in Purkinje neurons and moderate loss of large principal neurons could underlie cerebellar dysfunction and ataxia in this FRDA model, providing novel pathogenic mechanisms and potential therapeutic targets for treatment of FRDA patients.

## MATERIALS AND METHODS

### Materials

C57BL/6 mice were from Charles River Laboratories and frataxin KIKO mice were from the Jackson Laboratory (B6. Cg-Fxn^tm1.1Pand^ Fxn^tm1Mkn^/J; stock number 012329). KIKO mice were twice crossbred with Thy1-mitoDendra mice [B6SJL-Tg (Thy1-COX8A/Dendra)57Gmnf/J; stock number 025401] to generate control-mitoDendra and KIKO-mitoDendra mice (Magrané et al., 2014). All animals were treated according to the protocols approved by The Children's Hospital of Philadelphia Institutional Animal Care and Use Committee and Weill Cornell Medical College Institutional Animal Care and Use Committee. Antibodies included anti-GAD65 [Developmental Studies Hybridoma Bank, GAD-6, mouse monoclonal, 1:50 for immunohistochemistry (IHC)], anti-GAD65/67 [Millipore, AB1511, 1:1000 for western blotting (WB)], anti-VGLUT1 (Synaptic Systems, 135302, 1:1000 for IHC and 1:2000 for WB; Synaptic Systems, 135311, 1:500 for IHC), anti-VGLUT2 (Synaptic Systems, 135402, 1:1000 for WB), anti-frataxin (Abcam, ab175402, 1:250 for IHC and 1:1000 for WB), anti-PV (Millipore, AB1572, 1:400 for IHC and 1:1000 for WB; Swant, PV27, rabbit polyclonal, 1:1000 for IHC and WB), anti-ATP5A (MitoSciences, MS507, 1:500 for IHC), anti-GAPDH (Novus Biologicals, 1D4, NB300-221, 1:1000 for WB) and anti-actin [Abcam, ACTN5(04), ab3280, 1:5000 for WB] (Lin et al., 2014a,b, 2010).

### Tissue preparation and immunohistochemistry

For tissue homogenate preparation, cerebella of KIKO mice and age-matched heterogeneous controls [both wild-type/wild-type (WTWT) and knock-in/wild-type (KIWT) mice] at 1, 3, 6, and 9 months, which represent approximately postnatal days P30, P90, P180 and P270±10 days, of either sex were harvested. KIWT mice are the equivalent to human heterozygous carriers. The tissues were mechanically homogenized in 20 ml lysis buffer per 1 g weight, and lysed for 1 h at 4°C. Lysis buffer contained 150 mM NaCl, 1 mM EDTA, 100 mM Tris-HCl, 1% Triton X-100, and 1% sodium deoxycholate, pH 7.4, supplemented the day of use with 1:500 EDTA-free protease inhibitor cocktail III (Calbiochem, 53914). Debris was cleared by centrifugation at 39,000 ***g*** for 1 h at 4°C. Supernatants were stored at −80°C until use.

For immunohistochemical studies, KIKO-mitoDendra mice and age-matched KOWT-mitoDendra controls of either sex at P90 were perfused with 4% paraformaldehyde and their cerebella were harvested. KOWT mice were used as controls and are equivalent to the hemizygous carriers. A series of brain coronal floating sections (50 µm thick) was obtained using a vibratome (VT1200S; Leica, Deerfield, IL) in PBS; the sections were stored in PBS with 30% glycerol (vol/vol) and 30% ethylene glycerol (vol/vol) at −20°C. Floating sections were blocked with 5% normal goat serum and 1% bovine serum albumin in combination with 0.3% (vol/vol) Triton X-100 in PBS at room temperature for 1 h, then incubated with primary antibodies at 4°C overnight and then secondary antibodies conjugated to Alexa Fluor 488 (Invitrogen, A11029) or Alexa Fluor 568 (Invitrogen, A11036) at room temperature for 60-90 min. Following several washes with PBS, the stained sections were mounted on slides with Vectashield with DAPI (Vector Laboratories, H-1200).

### Transmission electron microscopy

For the ultrastructural studies, KIKO mice and age-matched KIWT controls of either sex at P150 and P360 (*n*=2 per group) were perfused with 2.5% glutaraldehyde and 2% paraformaldehyde and their cerebella were harvested. Tissues for electron microscopic examination were fixed with 2.5% glutaraldehyde, 2.0% paraformaldehyde in 0.1 M sodium cacodylate buffer, pH 7.4, overnight at 4°C. After subsequent buffer washes, the samples were postfixed in 2.0% osmium tetroxide for 1 h at room temperature, and then washed again in buffer followed by distilled H_2_O. After dehydration through a graded ethanol series, the tissue was infiltrated and embedded in EMbed-812 (Electron Microscopy Sciences, Fort Washington, PA). Thin sections were stained with uranyl acetate and lead citrate and examined with a JEOL 1010 electron microscope (JEOL USA, Peabody, MA) fitted with a Hamamatsu digital camera and AMT Advantage image capture software. Sample processing and staining were performed at the Electron Microscopy Resource Laboratory at University of Pennsylvania Perelman School of Medicine Biomedical Research Core Facilities.

### Western blot analysis

Western blotting was performed as described previously (Lin et al., 2014b). Protein content was determined using the BCA Protein Assay kit (Thermo Fisher Scientific). Equal amounts of total protein (30 µg tissue homogenate per lane) were subjected to 4-12% NuPAGE Gel for electrophoresis and transferred to nitrocellulose membranes. Membranes were blocked with 3% nonfat milk and incubated with primary antibody overnight at 4°C. Blots were then incubated with appropriate horseradish peroxidase (HRP)-conjugated secondary antibodies (Cell Signaling Technology) for 2 h at room temperature and then washed; reaction bands were visualized using a luminol-enhanced chemiluminescence (ECL) HRP substrate (Thermo Fisher Scientific). Each blot was then incubated with stripping buffer (2% SDS, 50 mM Tris, pH 6.8, and 100 mM β-mercaptoethanol) for 45 min at room temperature to remove the signals and reprobed for other proteins, including actin or GAPDH as an internal control. Reaction product levels were quantified by scanning densitometry using NIH ImageJ software (https://imagej.nih.gov/ij/) and normalized to the levels of actin and GAPDH.

### Immunohistochemistry

For immunohistochemistry, the floating sections were blocked with 5% normal goat serum and 1% bovine serum albumin in combination with 0.3% (vol/vol) Triton X-100 in PBS at room temperature for 1 h, then incubated with primary antibodies at 4°C overnight and then secondary antibodies conjugated to Alexa Fluor 488 or 594 (Invitrogen) at room temperature for 60-90 min. Following several washes with PBS, the stained sections were mounted on slides using Vectashield with DAPI.

### Fluorescence imaging and quantification

Fluorescence images were obtained with an Olympus FluoView or Leica SP8 laser scanning confocal microscope. Confocal scans were performed in mouse cerebellum, and the imaging parameters were identical for KIKO mice and knockout/wild-type (KOWT) controls. KOWT mice are the equivalent of hemizygous carriers. Control sections were included in all experiments to normalize for expected variations in antibody staining intensity performed on different days. The confocal images were acquired at the focal plane with a maximal number of VGLUT1-positive puncta or large principal neurons from three sections per animal, and from two to three animals per group. ImageJ software was used to quantify the number of VGLUT1-positive puncta in the cerebellar cortex or large principal neurons in the DN in the acquired confocal images. Thresholds were set at three standard deviations above the mean staining intensity of six nearby regions in the same visual field. Thresholded images present a fixed intensity for all pixels above threshold after having removed all of those below, and VGLUT1-positive puncta in the thresholded images were quantified (Lin et al., 2014b; Lozada et al., 2012).

### Statistical analysis

Data are shown as mean±s.e.m. Experimental results were analyzed using two-tailed, unpaired Student's *t*-test to compare two conditions. Significance was set at *P*<0.05.

## Supplementary Material

Supplementary information
