# Supplementary material for: Early VGLUT1-specific parallel fiber synaptic deficits and dysregulated cerebellar circuit in the KIKO mouse model of Friedreich ataxia
Source: Dis Model Mech. 2017 Dec 1;10(12):1529–38. doi: 10.1242/dmm.030049 (PMC5769605; doi:10.1242/dmm.030049)
Supplement: Supplementary information [file dmm-10-030049-s1.pdf]

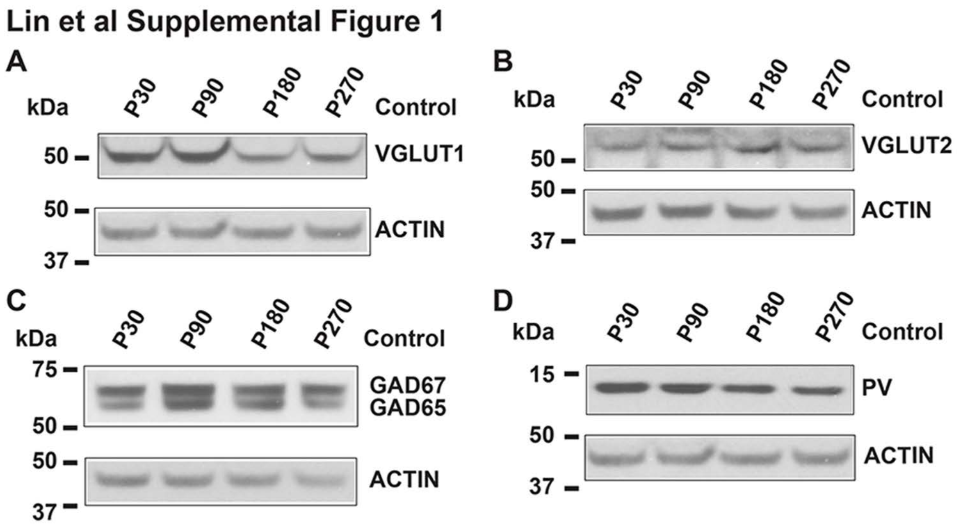

Supplemental Figure 1. VGLUT1, VGLUT2, GAD65/67 and PV expression in cerebellum of P30-P270 wild-type control mice. Western blotting of cerebellar homogenates (30 µg per lane) showing VGLUT1 (**A**), VGLUT2 (**B), GAD65/67 (C)** and PV (**D**) levels as well as actin as an internal control in the cerebellum of wild-type controls at postnatal days P30, P90, P180, and P270 (n=5-8 mice per time-point). For Figure **A** and **D**, blots were stripped and reprobed with α-VGLUT1 and α-PV antibodies; α-actin serves as the loading control for each.

Disease Models & Mechanisms • Supplementary information

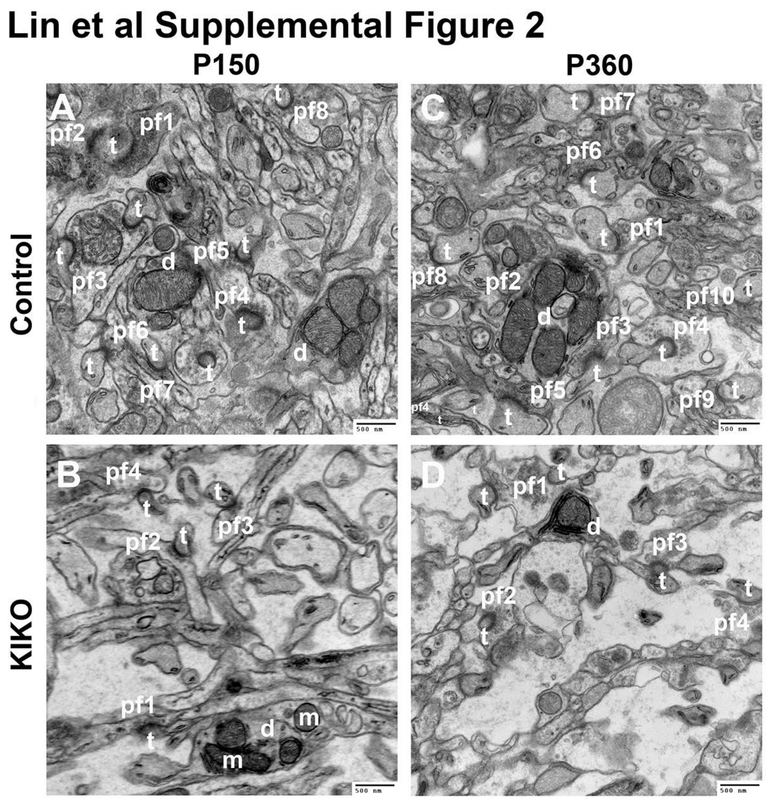

Supplemental Figure 2 Impaired ultrastructures of cerebellar molecular layer and parallel fiber synaptic decifits on Purkinje neurons in potentailly symptomatic and asymptomaic KIKO mice. Electron microscopy (EM) images of cerebellar molecular layer in asymptomatic (P150) and potentially symptomatic (P360) KIKO mice (**B**, **D**) compared with control (**A**, **C**). KIKO mice show impaired ultrastuctures of cerebellar molecular layer with enlarged empty spcae as well as fewer and smaller mitochondria in the dendritic branches (d) of Pukinje neurons of as well as decreased parallel fiber (PF) presynaptic terminals (pf) on the thorns (t) of Pukinje neuron dendritic branches in P150 (**B**) and P360 KIKO (**D**) mice compared with age-matched control mice (**A**, **C**). Scale bars as indicated.

Disease Models & Mechanisms • Supplementary information
